# Risk factors for the development of complications following blunt chest trauma: a new risk stratification tool

**DOI:** 10.1186/cc12230

**Published:** 2013-03-19

**Authors:** C Battle, H Hutchings, S Lovett, P Evans

**Affiliations:** 1 Morriston Hospital, ABMU Health Board, Swansea, UK; 2 Swansea University, Swansea, UK

## Introduction

The aim of the study was to investigate the risk factors for the development of complications following blunt chest trauma and to develop a risk stratification tool to assist in the management of this patient group. The difficulties in the management of this patient group in the emergency department (ED) due to the development of late complications are well recognised in the literature [1].

## Methods

Between 2009 and 2011 a total of 276 patients were admitted to hospital from the ED of a regional trauma centre in Wales, with the primary diagnosis of blunt chest trauma. Patients with immediate life-threatening injuries were excluded. Data were collected retrospectively and included risk factors (age, number of rib fractures, comorbidity, pre-injury anticoagulant use, smoking status, oxygen saturations and respiratory rate on initial assessment in the ED), and outcomes (mortality, any pulmonary morbidity, length of stay of 7 days or more and need for ICU admission). Development of complications was defined as the occurrence of one or more of the outcomes investigated. Multivariable logistic regression using fractional polynomials was used to identify risk factors and develop a risk stratification tool. The significant risk factors in the model were selected using backward elimination with the Akaike Information Criterion (AIC) at a significance level of 0.05. The *c *index and the Hosmer-Lemeshow (H-L) test were calculated to assess discrimination and calibration of the risk stratification tool respectively.

## Results

A total of 161 patients out of the 276 admitted developed complications following blunt chest trauma. Implementation of backward elimination using AIC values resulted in a final model based on the significant risk factors; age, oxygen saturations, number of rib fractures, presence of chronic lung disease and pre-injury anticoagulant use (all *P <*0.05). The *c *index for the tool was 0.80 and the H-L score was 9.22 (*P *= 0.32), indicating good predictive capabilities of the tool.

## Conclusion

The results of this study highlight the risk factors for the development of complications following blunt chest trauma. A risk stratification tool has also been developed that could assist in the prediction of poor outcomes in this patient group. The next stage is to complete a prospective validation study.
